# Current Applications, Opportunities, and Limitations of AI for 3D Imaging in Dental Research and Practice

**DOI:** 10.3390/ijerph17124424

**Published:** 2020-06-19

**Authors:** Kuofeng Hung, Andy Wai Kan Yeung, Ray Tanaka, Michael M. Bornstein

**Affiliations:** 1 Oral and Maxillofacial Radiology, Applied Oral Sciences and Community Dental Care, Faculty of Dentistry, The University of Hong Kong, Hong Kong 999077, China; hungkf@connect.hku.hk (K.H.); ndyeung@hku.hk (A.W.K.Y.); rayt3@hku.hk (R.T.); 2 Department of Oral Health & Medicine, University Center for Dental Medicine Basel UZB, University of Basel, 4058 Basel, Switzerland

**Keywords:** artificial intelligence, AI, machine learning, ML, cone beam computed tomography (CBCT), intraoral scanning, facial scanning

## Abstract

The increasing use of three-dimensional (3D) imaging techniques in dental medicine has boosted the development and use of artificial intelligence (AI) systems for various clinical problems. Cone beam computed tomography (CBCT) and intraoral/facial scans are potential sources of image data to develop 3D image-based AI systems for automated diagnosis, treatment planning, and prediction of treatment outcome. This review focuses on current developments and performance of AI for 3D imaging in dentomaxillofacial radiology (DMFR) as well as intraoral and facial scanning. In DMFR, machine learning-based algorithms proposed in the literature focus on three main applications, including automated diagnosis of dental and maxillofacial diseases, localization of anatomical landmarks for orthodontic and orthognathic treatment planning, and general improvement of image quality. Automatic recognition of teeth and diagnosis of facial deformations using AI systems based on intraoral and facial scanning will very likely be a field of increased interest in the future. The review is aimed at providing dental practitioners and interested colleagues in healthcare with a comprehensive understanding of the current trend of AI developments in the field of 3D imaging in dental medicine.

## 1. Introduction

Artificial intelligence (AI) is generally defined as intelligent computer programs capable of learning and applying knowledge to accomplish complex tasks such as to predict treatment outcomes, recognize objects, and answer questions [1]. Nowadays, AI technologies are widespread and penetrate many applications of our daily life, such as Amazon’s online shopping recommendations, Facebook’s image recognition, Netflix’s streaming videos, and the smartphone’s voice assistant [2]. For such daily life applications, it is characteristic that the initial use of an AI-driven system will give a more generalized outcome based on big data, and after repeated use by the individual, it will gradually present a more adapted and personalized outcome in accordance with the user’s characteristics. The remarkable success of AI in various fields of our daily life has inspired and is stimulating the development of AI systems in the field of medicine and, also, more specifically, dental medicine [3,4].

Radiology is deemed to be the front door for AI into medicine as digitally coded diagnostic images are more easily translated into computer language [5]. Thus, diagnostic images are seen as one of the primary sources of data used to develop AI systems for the purpose of an automated prediction of disease risk (such as osteoporotic bone fractures [6]), detection of pathologies (such as coronary artery calcification as a predictor for atherosclerosis [7]), and diagnosis of disease (such as skin cancers in dermatology [8]). Machine learning is a key component of AI, and commonly applied to develop image-based AI systems. Through a synergism between radiologists and the medical AI system used, increased work efficiency and more precise outcomes regarding the final diagnosis of various diseases are expected to be achieved [9,10].

In the field of dental and maxillofacial radiology (DMFR), reports on AI models used for diagnostic purposes and treatment planning cover a wide range of clinical applications, including automated localization of craniofacial anatomical structures/pathological changes, classification of maxillofacial cysts and/or tumors, and diagnosis of caries and periodontal lesions [11]. According to the literature related to clinical applications of AI in DMFR, most of the proposed machine learning algorithms were developed using two-dimensional (2D) diagnostic images, such as periapical, panoramic, and cephalometric radiographs [11]. However, 2D images have several limitations, including image magnification and distortion, superimposition of anatomical structures, and the lack of three-dimensional information for relevant landmarks/pathological changes. These may lower the diagnostic accuracy of the AI models trained using only 2D images [12]. For example, a 2D image-based AI model built for the detection of periodontal bone defects might not be able to detect three-walled bony defects, loss of buccal/oral cortical bone plates, or bone defects around overlapping teeth. Three-dimensional (3D) imaging techniques, including cone beam computed tomography (CBCT), as well as intraoral and facial scanning systems, are increasingly used in dental practice. CBCT imaging allows for the visualization and assessment of bony anatomic structures and/or pathological changes in 3D with high diagnostic accuracy and precision. The use of CBCT is of great help when conventional 2D imaging techniques do not provide sufficient information for diagnosis and treatment planning purposes [13]. Intraoral and facial scanning systems are reported to be reproducible and reliable to capture 3D soft-tissue images that can be used for digital treatment planning systems [14,15]. CBCT and intraoral/facial scans are considered as an ideal data source for developing AI models to overcome the limitations of 2D image-based algorithms [12,15]. Thus, the aim of this review is to describe current developments and to assess the performance of AI models for 3D imaging in DMFR, as well as intraoral and facial scanning.

## 2. Current Use of AI for 3D Imaging in DMFR

A literature search was conducted using PubMed to identify all existing studies of AI applications for 3D imaging in DMFR and intraoral/facial scanning. The search was conducted without restriction on the publication period but was limited to studies in English. The keywords used for the search were combinations of terms including “artificial intelligence”, “AI”, “machine learning”, “deep learning”, “convolutional neural networks”, “automatic”, ”automated”, “three-dimensional imaging”, “3D imaging”, “cone beam computed tomography”, “CBCT”, “three-dimensional scan”, “3D scan”, “intraoral scan”, “intraoral scanning”, “facial scan”, “facial scanning”, and/or “dentistry”. Reviews, conference papers, and studies using clinical/nonclinical image data were eligible for the initial screening process. Initially, titles of the identified studies were manually screened, and subsequently, abstracts of the relevant studies were read to identify studies for further full-text reading. Furthermore, references of included articles were examined to identify further relevant articles. As a result, approximately 650 publications were initially screened, and 23 publications were eventually included in the present review for data extraction (details provided in Table 1 and Table 2).

The methodological quality of the included studies was evaluated using the assessment criteria proposed by Hung et al. [11]. For proposed AI models for diagnosis/classification of a certain condition, four studies [16,17,18,19] were rated as having a “high” or an “unclear” risk of concern in the domain of subject selection because the testing dataset only consisted of images from subjects with the condition of interest. With regard to the selection of reference standards, all studies were considered as “low” risk of concern as expert judgment and clinical or pathological examination was applied as the reference standard. Concerns regarding the risk of bias were relatively high in the domain of index test, as ten [16,17,20,21,22,23,24,25,26,27] of the included studies did not test their AI models on independent images unused for developing the algorithms.

Table 1 exhibits the included studies regarding the use of AI for 3D imaging in DMFR. These studies focused on three main applications, including automated diagnosis of dental and maxillofacial diseases [16,17,18,19,20,28,29,30,31,32], localization of anatomical landmarks for orthodontic and orthognathic treatment planning [21,22,33,34,35], and improvement of image quality [23,36].

### 2.1. Automated Diagnosis of Dental and Maxillofacial Diseases

The basic principle of the learning algorithms for diagnostic purposes is to explore associations between the input image and output diagnosis. Theoretically, a machine learning algorithm is initially built using hand-crafted detectors of image features in a predefined framework, subsequently trained with the training data, iteratively adapted to minimize the error at the output, and eventually tested with the unseen testing data to verify its validity [37]. Deep learning, a subset of machine learning, is able to automatically learn to extract relevant image features without the requirement of the manual design of image feature detectors, which is currently considered as the most suitable method to develop image-based diagnostic AI models [12]. 

**Figure 1:**
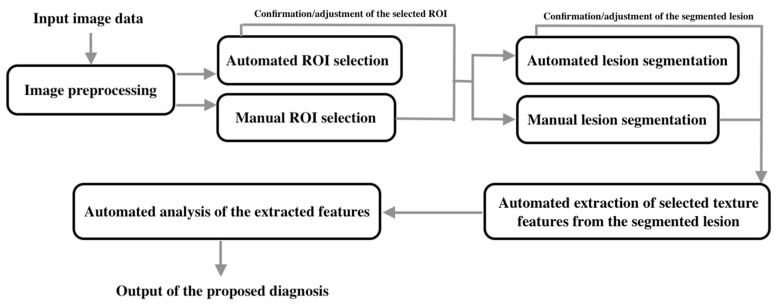
The workflow of the proposed machine learning algorithms for diagnostic purposes.

The workflow of the proposed machine learning algorithms for diagnostic purpose can be mainly categorized as (see Figure 1).

1. Input image data;
2. Image preprocessing;
3. Selection of the region of interest (ROI);
4. Segmentation of lesions;
5. Extraction of selected texture features in the segmented lesions;
6. Analysis of the extracted features;
7. Output of the diagnosis or classification.

Some of the proposed machine learning algorithms were not fully automated and required manual operation/adjustment for the ROI selection or lesion segmentation. Okada et al. proposed a semiautomatic machine learning algorithm, using CBCT images to classify periapical cysts and granulomas [16]. This algorithm requires users to segment the target lesion before it proceeds to the next step (feature extraction). Yilmaz et al. proposed a semiautomatic algorithm, using CBCT images to classify periapical cysts and keratocysts [18]. In this algorithm, detection and segmentation of lesions are required to be performed manually. The users need to mark the lesion on different cross-sectional planes to predefine the volume of interest containing the lesion. Manual segmentation of cystic lesions on multiple CBCT slices is time-consuming, which limits the efficiency of the algorithms and also their implementation for routine clinical use. Lee et al. proposed deep learning algorithms, respectively, using panoramic radiographs and CBCT images for the detection and diagnosis of periapical cysts, dentigerous cysts, and keratocysts [19]. It was reported that automatic edge detection techniques can segment cystic lesions more efficiently and accurately than manual segmentation. This can shorten the execution time for the segmentation step and improve the usability of the proposed algorithms for clinical practice. Moreover, higher diagnostic accuracy was reported for CBCT image-based algorithms in comparison with panoramic image-based ones. This may result from a higher accuracy in detecting the lesion boundary in 3D and more quantitative features extracted from the voxel units. Abdolali et al. proposed an algorithm based on asymmetry analysis using CBCT images to automatically segment cystic lesions, including dentigerous cysts, radicular cysts, and keratocysts [39]. The algorithm exhibited promising performance with high true-positives and low false-positives. However, its limitations include a relatively low detection rate for small cysts, imperfect segmentation of keratocysts without well-defined boundaries, and the incapability of dealing with symmetric cysts crossing the midsagittal plane. Based on the proposed segmentation algorithm, Abdolali et al. developed another AI model using CBCT images to automatically classify dentigerous cysts, radicular cysts, and keratocysts [17]. This model exhibited high classification accuracies ranging from 94.29% to 96.48%. Subsequently, Abdolali et al. further proposed a fully automated medical-content-based image retrieval system for the diagnosis of four maxillofacial lesions/conditions, including radiolucent lesions, maxillary sinus perforation, unerupted teeth, and root fractures [29]. In this novel system, an improved version of a previously proposed segmentation algorithm [39] was incorporated. The diagnostic accuracy of the proposed system was 90%, with a significantly reduced segmentation time of three minutes per case. It was stated that this system is more effective than previous models proposed in the literature, and is promising for introduction into clinical practice in the near future.

Orhan et al. verified the performance of a deep learning algorithm using CBCT images to detect and volumetrically measure periapical lesions [28]. A detection rate of 92.8% and a significant positive correlation between the automated and manual measurements were reported. The differences between manual and automated measurements are mainly due to inaccurate lesion segmentation. Because of low soft-tissue contrast in CBCT images, the deep learning algorithm exhibits difficulties in perfectly distinguishing the lesion area from neighboring soft tissue when buccal/oral cortical perforations or endo-perio lesions occur. Johari et al. proposed deep learning algorithms using periapical and CBCT images to detect vertical root fractures [30]. The results showed that the proposed model resulted in higher diagnostic performance for CBCT images than periapicals. Furthermore, some studies have reported on the application of deep learning algorithms for the diagnosis of Sjögren’s syndrome or lymph node metastasis. Kise et al. proposed a deep learning algorithm using CT images to assist inexperienced radiologists to semiautomatically diagnose Sjögren’s syndrome [32]. The results exhibited that the diagnostic performance of the deep learning algorithm is comparable to experienced radiologists and is significantly higher than for inexperienced radiologists. The main limitation of the proposed algorithm is its semiautomatic nature, requiring manual image segmentation prior to performing automated diagnosis. For further ease and implementation in daily routine, a completely automated segmentation of the region of the parotid gland should be developed and incorporated into a fully automated diagnostic system. Kann et al. and Ariji et al., respectively, proposed deep learning algorithms using contrast-enhanced CT images to semiautomatically identify nodal metastasis in patients with oral/head and neck cancer [20,31]. The user of the respective programs is required to manually segment the contour of lymph nodes on multiple CT slices. Excellent performance was reported for both algorithms proposed, which was close to or even surpassed the diagnostic accuracy of experienced radiologists. Therefore, these deep learning algorithms have the potential to help guide oral/head and neck cancer patient management. Future investigations should focus on the development of a fully automated identification system to avoid manual segmentation of lymph nodes. This can significantly improve the efficiency of the AI system used and could enable wider use of this system in community clinics.

### 2.2. Automated Localization of Anatomical Landmarks for Orthodontic and Orthognathic Treatment Planning

The correct analysis of craniofacial anatomy and facial proportions is the basis of successful orthodontic and orthognathic treatment. Traditional orthodontic analysis is generally conducted on 2D cephalometric radiographs, which can be less accurate due to image magnification, superimposition of structures, inappropriate X-ray projection angle, and patient position. Since CBCT was introduced in dental medicine, 3D diagnosis and virtual treatment planning have been assessed as a more accurate option for orthodontic and orthognathic treatment [40]. Although 3D orthodontic analysis can be performed by a computer-aided digital tracing approach, it still requires orthodontists to manually locate anatomical landmarks on multiple CBCT slices. The manual localization process is tedious and time-consuming, which may currently discourage orthodontists from switching to a fully digital workflow. Cheng et al. proposed the first machine learning algorithm to automatically localize one key landmark on CBCT images and reported promising results [33]. Subsequently, a series of machine learning algorithms were developed for automated localization of several anatomical landmarks and analysis of dentofacial deformity. Shahidi et al. proposed a machine-learning algorithm to automatically locate 14 craniofacial landmarks on CBCT images, whereas the mean deviation (3.40 mm) for all of the automatically identified landmarks was higher than the mean deviation (1.41 mm) for the manually detected ones [34]. Montufar et al. proposed two different automatic landmark localization systems, respectively, based on active shape models and a hybrid approach using active shape models followed by a 3D knowledge-based searching algorithm [21,22]. The mean deviation (2.51 mm) for all of the automatically identified landmarks in the hybrid system was lower than that of the system only using active shape models (3.64 mm). Despite less localization deviation, the performance of automated localization in the proposed systems is still not accurate enough to meet clinical requirements. Therefore, the existing AI systems can only be recommended for the use of preliminary localization of the orthodontic landmarks, but manual correction is still necessary prior to further orthodontic analyses. This may be the main limitation of these AI systems and this needs to be improved for future clinical dissemination and use.

Orthodontic and orthognathic treatments in patients with craniofacial deformities are challenging. The aforementioned AI systems may not be able to effectively deal with such patients. Torosdagli et al. proposed a novel deep learning algorithm applied for fully automated mandible segmentation and landmarking in craniofacial anomalies on CBCT images [35]. The proposed algorithm allows for orthodontic analysis in patients with craniofacial deformities and showed excellent performance with a sensitivity of 93.42% and specificity of 99.97%. Future studies should consider widening the field of applications for AI systems, especially for different patient populations.

### 2.3. Automated Improvement of Image Quality

Radiation dose protection is of paramount importance in medicine and also for DMFR. It is reported that medical radiation exposure is the largest artificial radiation source and represents approximately 14% of the total annual dose of ionizing radiation for individuals [41]. Computed tomography (CT) imaging is widely used to assist clinical diagnosis in various fields of medicine. Reducing the scanning slice thickness is the general option to enhance the resolution of CT images. However, this will increase the noise level as well as radiation dose exposure to the patient. High-resolution CT images are recommended only when low-resolution CT images do not provide sufficient information for diagnosis and treatment planning purposes in individual cases [42]. The balance between the radiation dose and CT image resolution is the biggest concern for radiologists. To address this issue, Park et al. proposed a deep learning algorithm to enhance the thick-slice CT image resolution similar to that of a thin slice [36]. It is reported that the noise level of the enhanced CT images is even lower than the original images. Therefore, this algorithm has the potential to be a useful tool for enhancing the image resolution for CT scans as well as reducing the radiation dose and noise level. It is expected that such an algorithm can further be developed for CBCT scans.

The presence of metal artifacts in CT/CBCT images is another critical issue that can obscure neighboring anatomical structures and interfere with disease diagnosis. In dental medicine, metal artifacts are not uncommon in CBCT images due to materials used for dental restorations or orthodontic purposes. These metal artifacts not only interfere with disease diagnosis but, in some cases, impede the image segmentation of the teeth and bony structures in the maxilla and mandible for computer-guided treatment. Minnema et al. proposed a deep learning algorithm based on a mixed-scale dense convolutional neural network for the segmentation of teeth and bone on CBCT images affected by metal artifacts [23]. It is reported that the proposed algorithm can accurately classify metal artifacts as background and segment teeth and bony structures. The promising results prove that a convolutional neural network is capable of extracting the characteristic features in CBCT voxel units that cannot be distinguished by human eyes.

### 2.4. Other Applications

In addition to the above AI applications, automated tooth detection, classification, and numbering are also fields of great interest, and they have the potential to simplify the process of filling out digital dental charts [43]. Miki et al. developed a deep learning algorithm based on a convolutional neural network to automatically classify tooth types based on CBCT images [38]. Although this algorithm was designed for automated filling of dental charts for forensic identification purposes, it may also be valuable to incorporate it into the digital treatment planning system, especially for use in implantology and prosthetics. For example, such an application may contribute to the automated identification of missing teeth for the diagnosis and planning of implants or other prosthetic treatments.

## 3. Current Use of AI for Intraoral 3D Imaging and Facial Scanning

In recent years, computer-aided design and manufacturing (CAD/CAM) technology have been widely used in various fields of dentistry, especially in implantology, prosthetics, orthodontics, and maxillofacial surgery. For example, CAD/CAM technology can be used for the fabrication of surgical implant guides, provisional/definitive restorations, orthodontic appliances, and maxillofacial surgical templates. Most of these applications are based on 3D hard and soft tissue images generated by CBCT and optical scanning (such as intraoral/facial scanning and scanning of dental casts/impressions). Intraoral scanning is the most accurate method of digitalizing the 3D contour of teeth and gingiva [44]. As a result, the intraoral scanning technique is now gradually replacing the scanning of dental casts or impressions and is also frequently used in CAD/CAM systems. Tooth segmentation is a critical step, which is usually performed manually by trained dental practitioners in a digital workflow to design and fabricate restorations and orthodontic appliances. However, manual segmentation is time-consuming, poorly reproducible, and limited due to human error, which may eventually have a negative influence on treatment outcome. Ghazvinian Zanjani et al. and Kim et al., respectively, developed deep learning algorithms for automated tooth segmentation on digitalized 3D dental surface models resulting in high segmentation precision (Table 2) [24,45]. These algorithms can speed up the digital workflow and reduce human error. Furthermore, Lian et al. proposed an automated tooth labeling algorithm based on intraoral scanning [25]. This algorithm can simplify the process of tooth position rearrangements in orthodontic treatment planning. 

Currently, only a few studies have reported on the use of machine learning techniques based on facial scanning (Table 2). Knoops et al. proposed an AI 3D-morphable model based on facial scanning to automatically analyze facial shape features for diagnosis and planning in plastic and reconstructive surgery [26]. In addition, this model is also able to predict patient-specific postoperative outcomes. The proposed model may improve the efficiency and accuracy in diagnosis and treatment planning, and help preoperative communication with the patient. However, this model can only perform an analysis based on 3D facial scanning alone. As facial scanning is unable to acquire volumetric bone data, the information about the underlying skeletal structures cannot be analyzed by this model. An updated model that can perform the analysis simultaneously on facial soft tissue and skeletal structures will be more realistic and probably more effective for clinical use.

Interestingly, facial scanning techniques in combination with AI can also be used for the diagnosis of neurodevelopmental disorders, such as autism spectrum disorder (ASD). Liu et al. explored the possibility of using a machine learning algorithm based on facial scanning to identify ASD and showed promising results with an accuracy of 88.51% (Table 2) [27]. This algorithm could be a supportive tool for the screening and diagnosis of ASD in clinical practice.

## 4. Limitations of the Included Studies

While the AI models proposed in the included studies have shown promising performance, several limitations are worth noting, which may affect the reliability of the proposed models. First, most of the proposed AI models were developed using a small number of images collected from the same institution over one defined time period (see details in Table 1, Table 2 and Table 3). Additionally, some classification models were only trained and tested using images from subjects with confirmed diseases (Table 3). These limitations might result in a risk of overfitting and a too optimistic appraisal of the proposed models. In addition, the images used to develop the algorithms might very likely be captured using the same device and imaging protocols, resulting in a lack of data heterogeneity (Table 3). This might cause a lack of generalizability and reliability of the proposed models and can result in inferior performance in clinical practice settings due to differences in variables, including devices, imaging protocols, and patient populations [46]. Thus, these models may still need to be verified by using adequate heterogeneous data collected from different dental institutions prior to being transferred and implemented into clinical practice.

## 5. Conclusions

The AI models described in the included studies exhibited various potential applications for 3D imaging in dental medicine, such as automated diagnosis of cystic lesions, localization of anatomical landmarks, and classification/segmentation of teeth (see details in Table 3). The performance of most of the proposed machine learning algorithms was considered satisfactory for clinical use, but with room for improvement. Currently, none of the algorithms described are commercially available. It is expected that the developed AI systems will be available as open-source for others to verify their findings and this will eventually lead to true impact in different dental settings. By such an approach, they will also be more easily accessible and potentially user-friendly for dental practitioners.

Up to date, most of the proposed machine learning algorithms were designed to address specific clinical issues in various fields of dental medicine. In the future, it is expected that various relevant algorithms would be integrated into one intelligent workflow system specifically designed for dental clinic use [47]. After input of the patient’s demographic data, medical history, clinical findings, 2D/3D diagnostic images, and/or intraoral/facial scans, the system could automatically conduct an overall analysis of the patient. The gathered data might contribute to a better understanding of the health condition of the respective patient and the development of personalized dental medicine, and subsequently, an individualized diagnosis, recommendations for comprehensive interdisciplinary treatment plans, and prediction of the treatment outcome and follow-up. This information will be provided to assist dental practitioners in making evidence-based decisions for each individual based on a real-time up-to-date big database. Furthermore, the capability of deep learning to analyze the information in each pixel/voxel unit may help to detect early lesions or unhealthy conditions that cannot be readily seen by human eyes. The future goals of AI development in dental medicine can be expected to not only improve patient care and radiologist’s work but also surpass human experts in achieving more timely diagnoses. Long working hours and uncomfortable work environments may affect the performance of radiologists, whereas a more consistent performance of AI systems can be achieved regardless of working hours and conditions.

It is worth noting that although the development of AI in healthcare is vigorously supported by world-leading medical and technological institutions, the current evidence of AI applications for 3D imaging in dental medicine is very limited. The lack of adequate studies on this topic has resulted in the present methodological approach to provide findings from the literature rather than a pure systematic review. Thus, a selection bias could very likely not be eliminated due to the design of the study, which is certainly a relevant limitation of the present article. Nevertheless, the results presented might have a positive and stimulating impact on future studies and research in this field and hopefully will result in academic debate.

## Figures and Tables

**Table 1:** Characteristics of studies describing machine learning-based artificial intelligence (AI) models applied in dentomaxillofacial radiology (DMFR).

Author (Year)	Application	Imaging Modality	AI Technique	Image Data Set Used to Develop the AI Model	Independent Testing Image Data Set / Validation Technique	Performance
Diagnosis of Dental and Maxillofacial Diseases						
Okada [16] (2015)	Diagnosis of periapical cysts and granuloma	CBCT	LDA	28 scans from patients with periapical cysts or granuloma	7-fold CV	94.1% (accuracy)
Abdolali [17] (2017)	Diagnosis of radicular cysts, dentigerous cysts, and keratocysts	CBCT	SVM; SDA	96 scans from patients with radicular cysts, dentigerous cysts, or keratocysts	3-fold CV	94.29–96.48% (accuracy)
Yilmaz [18] (2017)	Diagnosis of periapical cysts and keratocysts	CBCT	k-NN; Naïve Bayes; Decision tree; Random forest; NN; SVM	50 scans from patients with cysts or tumors	10-fold CV/LOOCV	94–100% (accuracy)
				25 scans from patients with cysts or tumors	25 scans from patients with cysts or tumors	
Lee [19] (2020)	Diagnosis of periapical cysts, dentigerous cysts, and keratocysts	Panoramic radiography and CBCT	CNN	912 panoramic images and 789 CBCT scans	228 panoramic images and 197 CBCT scans	Panoramic radiography 0.847 (AUC); 88.2% (sensitivity); 77.0% (specificity) CBCT 0.914 (AUC); 96.1% (sensitivity); 77.1% (specificity)
Orhan [28] (2020)	Diagnosis of periapical pathology	CBCT	CNN	3900 scans acquired using multiple FOVs from 2800 patients with periapical lesions and 1100 subjects without periapical lesions	109 scans acquired using multiple FOVs from 153 patients with periapical lesions	92.8% (accuracy)
Abdolali [29] (2019)	Diagnosis of radiolucent lesion, maxillary sinus perforation, unerupted tooth, and root fracture	CBCT	Symmetry-based analysis model	686 scans acquired using a large FOV (12 × 15 × 15 cm^3^), collected from several dental imaging centers in Iran	459 scans acquired using a large FOV (12 × 15 × 15 cm^3^), collected from several dental imaging centers in Iran	0.85–0.92 (DSC)
Johari [30] (2017)	Detection of vertical root fractures	Periapical radiography and CBCT	CNN	180 periapical radiographs and 180 CBCT scans of the extracted teeth	60 periapical radiographs and 60 CBCT scans of the extracted teeth	Periapical radiography 70.0% (accuracy); 97.8% (sensitivity); 67.6% (specificity) CBCT 96.6% (accuracy); 93.3% (sensitivity); 100% (specificity)
Kise [32] (2019)	Diagnosis of Sjögren’s syndrome	CT	CNN	400 scans (200 from 20 SjS patients and 200 from 20 control subjects) acquired using a large FOV	100 scans (50 from 5 SjS patients and 50 from 5 control subjects) acquired using a large FOV	96.0% (accuracy); 100% (sensitivity); 92.0% (specificity)
Kann [31] (2018)	Detection of lymph node metastasis and extranodal extension in patients with head and neck cancer	Contrast-enhanced CT	CNN	Images of 2875 CT-segmented lymph node samples with correlating pathology labels	Images of 131 lymph nodes (76 negative and 55 positive)	0.91 (AUC)
Ariji [20] (2019)	Detection of lymph node metastasis in patients with oral cancer	Contrast-enhanced CT	CNN	Images of 441 lymph nodes (314 negative and 127 positive) from 45 patients	5-fold CV	78.2% (accuracy); 75.4% (sensitivity); 81.0% (specificity), 0.80 (AUC)
Localization of Anatomical Landmarks for Orthodontic and Orthognathic Treatment Planning						
Cheng [33] (2011)	Localization of the odontoid process of the second vertebra	CBCT	Random forest	50 scans	23 scans	3.15 mm (mean deviation)
Shahidi [34] (2014)	Localization of 14 anatomical landmarks	CBCT	Feature-based and voxel similarity-based algorithms	8 scans acquired using a large FOV from subjects aged 10–45 years	20 scans acquired using a large FOV from subjects aged 10–45 years	3.40 mm (mean deviation)
Montufar [21] (2018)	Localization of 18 anatomical landmarks	CBCT	Active shape model	24 scans acquired using a large FOV	LOOCV	3.64 mm (mean deviation)
Montufar [22] (2018)	Localization of 18 anatomical landmarks	CBCT	Active shape model	24 scans acquired using a large FOV	LOOCV	2.51 mm (mean deviation)
Torosdagli [35] (2019)	Localization of 9 anatomical landmarks	CBCT	CNN	50 scans	48 scans	0.9382 (DSC); 93.42% (sensitivity); 99.97% (specificity),
Improvement of Image Quality						
Park [36] (2018)	Improvement of image resolution	CT	CNN	52 scans	13 scans	The CNN network can yield high-resolution images based on low-resolution images
Minnema [23] (2019)	Segmentation of CBCT scans affected by metal artifacts	CBCT	CNN	20 scans	Leave-2-out CV	The CNN network can accurately segment bony structures in CBCT scans affected by metal artifacts
Other						
Miki [38] (2017)	Tooth classification	CBCT	CNN	42 scans with the diameter of the FOV ranged from 5.1 to 20 cm	10 scans with the diameter of the FOV ranged from 5.1 to 20 cm	88.8% (accuracy)

AI, artificial intelligence; AUC, area under the receiver operating characteristic curve; CBCT, cone beam computed tomography; CNN, convolutional neural network; CT, computed tomography; CV, cross validation; DSC, dice similarity coefficient; FOV, field of view; k-NN, k-nearest neighbors; LDA, linear discriminant analysis; LOOCV, leave-one-out cross-validation; NN, neural network; SDA, sparse discriminant analysis; SjS, Sjögren’s syndrome; SVM, support vector machine.

**Table 2:** Characteristics of the machine learning-based AI models based on intraoral and facial scanning.

Author (Year)	Application	Imaging Modality	AI Technique	Image Data Set Used to Develop the AI Model	Independent Testing Image Data Set/Validation Technique	Performance
Ghazvinian Zanjani [24] (2019)	Tooth segmentation	Intraoral scanning	CNN	120 scans, comprising 60 upper jaws and 60 lower jaws.	5-fold CV	0.94 (intersection over union score)
Kim [45] (2020)	Tooth segmentation	Intraoral scanning	Generative adversarial network	10,000 cropped images	Approximate 350 cropped images	An average improvement of 0.004 mm in the tooth segmentation
Lian [25] (2020)	Tooth labelling	Intraoral scanning	CNN	30 scans of upper jaws	5-fold CV	0.894 to 0.970 (DSC)
Liu [27] (2016)	Identification of Autism Spectrum Disorder	Facial scanning	SVM	87 scans from children with and without Autism Spectrum Disorder	LOOCV	88.51% (accuracy)
Knoops [26] (2019)	Diagnosis and planning in plastic and reconstructive surgery	Facial scanning	Machine-learning-based 3D morphable model	4261 scans from healthy subjects and orthognathic patients	LOOCV	Diagnosis 95.5% (sensitivity); 95.2% (specificity) Surgical simulation 1.1 ± 0.3 mm (accuracy)

3D, three-dimensional; AI, artificial intelligence; CV, cross-validation; DSC, dice similarity coefficient; LOOCV, leave-one-out cross-validation; SVM, support vector machine.

**Table 3:** Conclusions and limitations of the included studies.

Author (Year)	Conclusion	Limitations (Risk of Bias *)
Okada [16] (2015)	The proposed model may assist clinicians to accurately differentiate periapical lesions.	A small training dataset *; Lacking data heterogeneity *; Dataset only consisted of scans from subjects with the condition of interest *; Lacking independent unseen testing data *; Manual ROI selection; Long execution time.
Abdolali [17] (2017)	The proposed model can improve the accuracy of the diagnosis of dentigerous cysts, radicular cysts, and keratocysts, and may have a significant impact on future AI diagnostic systems.	A small training dataset *; Lacking data heterogeneity *; Dataset only consisted of scans from subjects with the condition of interest *; Lacking independent unseen testing data *.
Yilmaz [18] (2017)	Periapical cysts and keratocysts can be classified with high accuracy with the proposed model. It can also contribute to the field of automated diagnosis of periapical lesions.	A small training dataset *; Lacking data heterogeneity *; Dataset only consisted of scans from subjects with the condition of interest *; Manual detection and segmentation of lesions.
Lee [19] (2020)	Periapical cysts, dentigerous cysts, and keratocysts can be effectively detected and diagnosed with the proposed deep CNN algorithm, but the diagnosis of these lesions using radiological data alone, without histological examination, is still challenging.	A relatively small training dataset *; Dataset only consisted of scans from subjects with the condition of interest *; Manual ROI selection; Potential overfitting problem in the training procedure *.
Orhan [28] (2020)	The proposed deep learning systems can be useful for detection and volumetric measurement of periapical lesions. The diagnostic performance was comparable to that of an oral and maxillofacial radiologist.	Relatively inaccurate segmentation of lesions in close contact with neighboring soft tissue
Abdolali [29] (2019)	The proposed system is effective and can automatically diagnose various maxillofacial lesions/conditions. It can facilitate the introduction of content-based image retrieval in clinical CBCT applications.	Relatively inaccurate detection of symmetric lesions
Johari [30] (2017)	The proposed deep learning model can be used for the diagnosis of vertical root fractures on CBCT images of endodontically treated and also vital teeth. With the aid of the model, the use of CBCT images is more effective than periapical radiographs.	A small training dataset *; Ex-vivo data only containing sound extracted premolars *; Lacking data heterogeneity *; Unknown diagnostic performance on multirooted teeth and teeth with caries or filling materials *.
Kise [32] (2019)	The deep learning model showed high diagnostic accuracy for SjS, which is comparable to that of experienced radiologists. It is suggested that the model could be used to assist the diagnosis of SjS, especially for inexperienced radiologists.	A small training dataset *; Lacking data heterogeneity *; Lacking subjects with other pathological changes of the parotid gland in the control subjects *; Manual ROI segmentation.
Kann [31] (2018)	The proposed deep learning model has the potential for use as a clinical decision-making tool to help guide head and neck cancer patient management.	The process of individual lymph node CT labeling in correlation with pathology reports is subject to some degree of uncertainty and subjectivity *; Only lymph nodes for which a definitive correlation could be made were included in the labeled dataset, potentially biasing the dataset to those nodes that could be definitively correlated with pathologic report *.
Ariji [20] (2019)	The proposed deep learning model yielded diagnostic results comparable to that of radiologists, which suggests that the model may be valuable for diagnostic support.	A small training dataset *; Lacking data heterogeneity *; Lacking independent unseen testing data *; Manual ROI segmentation;
Cheng [33] (2011)	The proposed model can efficiently assist clinicians in locating the odontoid process of the second vertebra.	A small training dataset *; Lacking data heterogeneity *; Inaccurate localization performance.
Shahidi [34] (2014)	The localization performance of the proposed model was acceptable with a mean deviation of 3.40 mm for all automatically identified landmarks.	A small training dataset *; Lacking data heterogeneity *; Inaccurate localization performance.
Montufar [21] (2018)	The proposed algorithm for automatically locating landmarks on CBCT volumes seems to be useful for 3D cephalometric analysis.	A small training dataset *; Lacking data heterogeneity *; Lacking independent unseen testing data *; Inaccurate localization performance.
Montufar [22] (2018)	The proposed hybrid algorithm for automatic landmarking on CBCT volumes seems to be potentially useful for 3D cephalometric analysis.	A small training dataset *; Lacking data heterogeneity *; Lacking independent unseen testing data *; Relatively inaccurate localization performance.
Torosdagli [35] (2019)	The proposed deep learning algorithm allows for orthodontic analysis in patients with craniofacial deformities exhibiting excellent performance.	A small training dataset *; Lacking data heterogeneity *; Analysis of pseudo-3D images instead of fully 3D images *;
Park [36] (2018)	The proposed deep learning algorithm is useful for super-resolution and de-noising.	A small training dataset *; Small anatomical structures may be easily buried and invisible in low-resolution images.
Minnema [23] (2019)	The proposed deep learning algorithm allows us to accurately classify metal artifacts as background noise, and to segment teeth and bony structures.	A small training dataset *; Lacking independent unseen testing data *; Potential bias in the overall accuracy of the gold standard segmentations *.
Miki [38] (2017)	The proposed deep learning algorithm to classify tooth types on CBCTs yielded a high performance. This can be effectively used for automated preparation of dental charts and might be useful in forensic identification.	A small training dataset *; Unstable classification performance due to the analyzed levels of the cross-sectional tooth images and metal artifacts;
Ghazvinian Zanjani [24] (2019)	The proposed end-to-end deep learning framework for the segmentation of individual teeth and the gingiva from intraoral scans outperforms state-of-the-art networks.	A small training dataset *; Ex-vivo data *; Lacking independent unseen testing data *;
Kim [45] (2020)	The proposed automated segmentation method for full arch intraoral scan data is as accurate as a manual segmentation method. This tool could efficiently facilitate the digital setup process in orthodontic treatment.	Ex-vivo data *; Unable to automatically detect the occlusion area.
Lian [25] (2020)	The proposed end-to-end deep neural network to automatically label individual teeth on raw dental surfaces acquired by 3D intraoral scanners outperforms the state-of-the-art methods for 3D shape segmentation.	A small training dataset *; Scans only containing the maxillary dental surfaces with the complete 14 teeth *; Failed to properly handle missing teeth and additional braces in challenging cases; Lacking independent unseen testing data *;
Liu [27] (2016)	The proposed machine learning algorithm based on face scanning patterns could support current clinical practice of the screening and diagnosis of ASD	A small training dataset *; Lacking independent unseen testing data *; Several influencing factors, such as age-/culture-adapted face scanning patterns and the characteristics of the ASD patients should be considered when applying the model to classify children with ASD *.
Knoops [26] (2019)	The proposed model can automatically analyze facial shape features and provide patient-specific treatment plans from a 3D facial scan. This may benefit the clinical decision-making process and improve clinical understanding of face shape as a marker for plastic and reconstructive surgery.	Lacking independent unseen testing data *

3D, three-dimensional; AI, artificial intelligence; ASD, autism spectrum disorder; CBCT, cone beam computed tomography; CT, computed tomography; CNN, convolutional neural network; ROI, region of interest; SjS, Sjögren’s syndrome; * risk of bias.
